# Bringing the hospital home: exploring the challenges and perspectives of healthcare professionals on home treatment in hemato-oncology: a qualitative analysis

**DOI:** 10.1007/s00520-026-10981-8

**Published:** 2026-07-23

**Authors:** Ester Eliyahu, Ariel Aviv, Adir Shaulov, Osnat Bairey, Noam Benyamini, Ilana Hellmann, Merav Leiba, Tsofia Levi, Tamar Tadmor, Miriam Khalaily Ayoub, Yana Douvdevany, Orna Baron-Epel

**Affiliations:** 1 School of Public Health, University of Haifa, Haifa, Israel; 2 Department of Hematology, Emek Medical Center, Afula, Israel; 3 Department of Hematology, Hadassah Medical Center and Faculty of Medicine, Hebrew University of Jerusalem, Jerusalem, Israel; 4 Department of Hematology, Rabin Medical Center, Beilinson Hospital, Petah Tikva, Israel; 5 Department of Hematology, Tzafon Medical Center, Poria, Israel; 6 Department of Hematology, Meir Medical Center, Kfar Saba, Israel; 7 Department of Hematology, Assuta Ashdod University Hospital, Ashdod, and Faculty of Health Sciences, Ben-Gurion University of the Negev, Beersheba, Israel; 8 Department of Hematology, Rambam Health Care Campus, Haifa, Israel; 9 Hematology Unit, Bnai Zion Medical Center, and the Ruth and Bruce Rappaport Faculty of Medicine, Technion, Haifa, Israel

**Keywords:** Home treatment, Hemato-oncology, Healthcare professionals, Clinical perspectives, Health policy

## Abstract

**Purpose:**

This study aimed to examine the perceived challenges and attitudes toward home-based treatment for hematology and hemato-oncology patients among physicians and nurses.

**Methods:**

A qualitative study was conducted based on 23 semistructured interviews with physicians (*n* = 11) and nurses (*n* = 12) from eight hospitals across Israel. The participants were recruited using opportunistic sampling, and the interviews were analyzed thematically using an inductive reflexive thematic analysis approach. Data saturation was achieved.

**Results:**

Three overarching themes emerged: (1) *the hospital as a safe haven*: *the conflict between hospital safety and the benefits of home treatment*; participants emphasized the importance of hospital-based monitoring and expressed concerns about patient safety in home settings; (2) *building a well-functioning home treatment system vs. fearing loss of control*; while envisioning an organized, hospital-led model, participants stressed the need for trained staff, dedicated coordination, and clinical oversight; and (3) *balancing costs, risks, and institutional support*; participants highlighted financial disincentives, infrastructure gaps, and the role of professional trust in promoting patient acceptance.

**Conclusions:**

Although healthcare professionals recognize the potential benefits of home-based treatment, their support is contingent upon robust clinical protocols, institutional alignment, and clear coordination mechanisms. These findings underscore the central role of provider engagement in advancing safe and sustainable models of home care for hematology and hemato-oncology patients. The study offers practical insights that will assist in implementing health policies, developing clinical programs, and organizational planning for the safe and sustainable implementation of nonpalliative home care in hematology and hemato-oncology.

**Supplementary information:**

The online version contains supplementary material available at 10.1007/s00520-026-10981-8.

## Introduction

Over the past decade, home care has emerged as a growing component of health services worldwide [1, 2], driven by growing pressure on hospital infrastructure, population aging, economic considerations, and patients’ preferences for receiving treatment in a familiar environment [2, 3].

Among patients with hematologic and hemato-oncologic malignancies, growing evidence indicates that a wide range of active treatments can be delivered in home settings across different stages of disease and among diverse patient groups [4]. Research has demonstrated its clinical feasibility, psychological benefits, and positive impact on patients’ quality of life [5–7]. Nevertheless, home chemotherapy presents unique challenges such as the need for multiple staff members to manage logistics, strict adherence to regulations, ensuring drug stability conditions [7, 8], ensuring treatment safety, and managing potential side effects. Accordingly, home-based chemotherapy requires careful patient selection, close monitoring, and comprehensive education for patients and their caregivers [9]. Nursing care plays a central role in home chemotherapy, including patient education, treatment monitoring, and side effect management, which contribute to maintaining the quality and safety of treatment [10]. However, a comprehensive evidence synthesis reported no consistent differences in clinical outcomes or quality of life between home, community, and hospital outpatient chemotherapy settings; instead, qualitative evidence indicated that decisions were largely influenced by perceptions of safety, professional support, and organizational context [11].

In Israel, a cross-sectional survey among transfusion-dependent hematology patients revealed that more than half expressed a preference for receiving transfusions at home rather than in hospital settings. Their willingness was closely linked to the perceived negative effect of hospital-based transfusion on quality of life, while concerns about adverse effects and loss of contact with the hospital staff were the main barriers [12]. These findings highlight patients’ openness to home-based options but also point out the need to explore how healthcare professionals themselves perceive these services.

Although preferences for home-based care have been increasingly documented among patients, studies suggest that both patients and healthcare professionals hold diverse and sometimes ambivalent views regarding this type of care. Healthcare professionals often acknowledge the clinical and humanistic value of treating patients at home and acknowledge its potential as an alternative to traditional hospitalization. At the same time, they express significant concerns related to safety, excessive workload, organizational fragmentation, lack of training, and insufficient organizational as well as legislative infrastructure [13, 14]. These challenges are especially apparent in the care of medically complex patients, where responsibility is often shared among multiple stakeholders across disconnected systems. In Israel, where acute medical home care is offered as an alternative to hospitalization in some internal medicine departments, most physicians surveyed viewed it as a valuable substitute for hospital-based care. However, several barriers to wider implementation were identified, including a shortage of specialized personnel, lack of economic resources, limited awareness of the service, and resistance among stakeholders, whether due to lack of interest or a conservative preference for traditional hospitalization models [1].

In recent years, home health services have expanded in Israel to include home hospitalization programs, home palliative care services, and selected treatments provided in patients' homes or in the community. Within hematology, certain treatments such as subcutaneous immunoglobulin administration are routinely administered in the home setting, and some supportive treatments, including intravenous iron administration, may be administered outside of hospital inpatient settings. However, routine provision of active hemato-oncology treatments in patients’ homes remains limited. As a result, the perspectives of healthcare professionals regarding the broader implementation of home hemato-oncology care remain particularly important.

Despite growing interest in home-based care, little is known about how it is perceived by healthcare professionals and patients in hematology and hemato-oncology. We focused on this subpopulation due to several reasons: 1) the relatively complex and sometimes unpredictable trajectory of hematological diseases, 2) the usually long-lasting relations between patients and hematology teams, and 3) the well-described “transfusion tether” tying many hematological patients to blood banks and hospitals [15].

In this study, the term “home treatments” was used broadly to refer to the possibility of transferring treatments and services currently provided in hospital hematology and hemato-oncology settings to patients’ homes. Interviews were not limited to a specific treatment modality, and participants discussed a variety of interventions that could be delivered at home, including blood transfusions, subcutaneous therapies, selected intravenous therapies, and supportive care services such as hydration and symptom management.

This study aims to examine the perceived challenges and attitudes toward home-based treatment for hematology and hemato-oncology patients among physicians and nurses, with the goal of generating practical insights to support the future design and implementation of such services in the Israeli healthcare system.

## Materials and methods

A qualitative study was conducted to explore the attitudes of physicians and nurses treating hematology and hemato-oncology patients in outpatient clinics within 8 hospitals in Israel. The study included qualitative interviews with healthcare providers regarding home care treatments for these patients. Ethical approval was obtained from the University of Haifa Ethics Committee (156/24), and the Helsinki Committee’s approval was received from each participating hospital. Verbal and written informed consent to be interviewed for the study was provided for all participants.

### Participants and recruitment

The study population included 11 physicians and 12 nurses from 8 hospitals in Israel. An opportunistic sampling approach was used, whereby staff members present during the shifts were invited to participate, justified by both the exploratory nature of the study and the ethical and logistical framework of the research. As this is an initial qualitative investigation into an underexplored topic, the aim was to capture a range of professional perspectives rather than achieve statistical representativeness. Despite the nonprobabilistic sampling method, the sample was diverse and included participants from all eight hospitals. Participating hospitals were selected to reflect the diversity of hematology services in the Israeli healthcare system. The sample included large care centers and peripheral hospitals, located in the north, center, south, and Jerusalem regions of the country. Hospitals also varied in their organizational affiliation, including both government-owned hospitals and hospitals operated by health maintenance organizations (HMOs). In addition, familiarity with home care models varied across institutions. While home hemato-oncology services were not routinely available in any of the participating hematology units, some hospitals had experience with home care programs in other medical fields, such as internal medicine, in which patients received treatments including intravenous antibiotics and fluid administration at home. This diversity was intended to capture a wide range of professional perspectives regarding the feasibility, challenges, and implementation of home-based hemato-oncology care. The interviewed physicians included both male (*n* = 4) and female (*n* = 7) doctors, ranging in seniority from residents to department chairs. The nursing staff included one male nurse and eleven female nurses. Their professional roles within the hematology day care units varied: some had been working in the units for several years and were still considered relatively new to the hematology field, while others held senior managerial positions as head nurses.

In each hospital, the local principal investigator, who was not necessarily the head of the department and could also be a fellow, approached the staff members present during the shift and informed them about the study. The invitation to participate was addressed generically to all staff members present, without tracking who agreed or refused. The interviewer was then introduced, and participation was completely voluntary. Three female interviewers conducted the interviews: the first author (EE), a PhD candidate in Public Health, and two trained research assistants with graduate-level training and prior experience in qualitative interviewing. None of the interviewers had any prior acquaintance or professional relationship with the participants. The interviews were intentionally designed to be short and technically focused, as they addressed specific professional, organizational, and implementation aspects of home treatments. Participants were able to provide concise and focused responses despite the relatively brief interview format.

### Data collection

The interviews were conducted using a semistructured interview guide developed by the research team to address the study’s objectives (see additional file 1). A total of 23 interviews were conducted. The interviews were professionally focused, addressing practical, organizational, and clinical–perceptual aspects of home treatment. Given the clinical workload and time constraints of the participants, the interviews were designed to be concise and focused, rather than emotionally oriented in-depth interviews. Data collection continued until thematic saturation was reached. Saturation was considered achieved when no new codes, concepts, or thematic insights emerged during the analysis process. After the analysis of the first 20 interviews, no substantive new themes were identified. Three additional interviews were conducted and analyzed to confirm saturation, and these interviews primarily reinforced existing themes rather than creating new thematic content. Twenty interviews were conducted face-to-face in a quiet and private room within the outpatient departments of the participating hospitals, while three interviews were conducted via Zoom due to logistical considerations. Interviews lasted between 8 and 35 min, with a mean duration of 21 min.

### Data analysis

Interviews were audio-recorded and transcribed verbatim. All interviews were conducted in Hebrew. The data were analyzed using a reflexive thematic analysis approach, following the principles outlined by Braun and Clarke [16]. The analysis was conducted iteratively, with constant movement between data, coding, and theme development. The researchers actively engaged in reflexive interpretation throughout the analytic process, recognizing their role in shaping the analysis. In the first stage, all interviews were read, after which the text was divided into meaningful categories. Coding was performed manually in the source language (Hebrew) under the first author’s leadership. Emerging categories and themes were regularly discussed with the research team (OBE, AA, and AS) throughout the analysis process. When different interpretations emerged, the researchers returned to the original transcripts, examined relevant passages, and considered alternative explanations until consensus was reached on the most appropriate representation of the data. The analysis was conducted under the close supervision of OBE, an experienced qualitative researcher, who reviewed coding decisions, challenged interpretations, and provided ongoing methodological guidance. Direct quotes from participants were used to support and illustrate the identified themes.

This study was conducted and reported in accordance with the Consolidated Criteria for Reporting Qualitative Research (COREQ) checklist. The complete COREQ checklist is provided as Supplementary Material (Additional file 2).

### Trustworthiness

To enhance the reliability of the study, several strategies were used. Research reliability was supported through semistructured interviews, which allowed participants to elaborate on their perceptions, and with direct quotes to ground findings in the data. In addition, the analysis process was reviewed by other members of the research team to ensure that the interpretation accurately reflected participants’ perspectives. Research reliability was addressed by maintaining a clear and documented audit trail of all stages of data collection and analysis, including a structured coding process. Reflexivity was maintained by ongoing awareness of the first author’s professional position and personal interest in promoting home care, and by ongoing reflection on how this position might influence data interpretation.

## Results

### Theme 1: the hospital as a safe haven: the conflict between hospital safety and the benefits of home treatment

#### The hospital as a stronghold of safety and clinical supervision

Physicians and nurses portrayed the hospital as a stronghold of safety and clinical oversight, the natural and most secure setting for administering treatments. They described the hospital not merely as a site of care, but as an essential protective environment where immediate response to adverse events is possible. The idea of shifting treatments to the patient’s home raised major concerns, especially regarding the potential for side effects to go unnoticed and the loss of continuous, hands-on clinical monitoring that hospital settings provide.

*If there is a medication that can cause side effects…then there is no doubt that it should be administered in a hospital setting. (Physician 8*).

*There’s something about when a patient arrives…there’s also that personal element. That's less likely to happen because you see the patient less.. (Physician 6)*

In hospital-based outpatient services, nurses maintain a more intensive relationship with patients, seeing them throughout treatment and enabling close monitoring of changes, while physicians see patients during prescheduled visits and sometimes during day hospitalization as they rotate between patients. Therefore, in their view, some patients may refuse home treatment due to a sense of security associated with hospital care, as emphasized by many physicians. One physician highlighted that interactions among patients in the hospital provide a sense of support and shared fate. Some of the nurses also addressed this issue, noting that it could pose a particular challenge, especially for patients with weak home support systems.

*I think it gives them a sense of security, that things are under control, that they see them doing tests… it gives them a sense of calm… to come here and see us. (Physician 9)*

*So those people who would most prefer to come here are people who don’t have a proper support system, people who are afraid to be alone. (Nurse 10)*

While all participants viewed the hospital as a safe and controlled environment for treatment, a small subset (3 out of 23) noted a specific advantage of home-based care in reducing the risk of hospital-acquired infections. These participants emphasized that hospitals, while offering clinical oversight, are also environments with high exposure to pathogens, including antibiotic-resistant bacteria. This issue was mentioned by only three participants, a relatively small number considering the high infection risk commonly associated with immunocompromised hemato-oncology patients.

*The hospital means it’s full of bacteria, and resistant bacteria, so if it’s possible to avoid going to the hospital, it’s better. (Nurse 1)*

#### Concerns about fragmentation, risk, and unmanageable complexity

Alongside the vision for an ideal home treatment system, interviewees identified several logistical and regulatory challenges that must be addressed to ensure patient safety and the feasibility of home-based care. The main logistical barriers included (1) medication preparation and stability, (2) ordering and transportation of blood products, (3) ensuring the availability of necessary equipment, (4) managing long or complex treatments that require prolonged staff presence or alternating interventions throughout the day, and (5) handling the administration of cytotoxic drugs.

*There are medications that break down quickly. If you prepare them, you need to administer them within twenty minutes, half an hour to an hour. These are treatments that are difficult to administer at home. (Nurse 11).*

These logistical barriers were perceived as particularly significant by nurses. While physicians acknowledged these obstacles, they generally regarded them as practical challenges that could be addressed through careful planning and system design. In contrast, many nurses expressed deeper concerns and skepticism, describing some of the issues as nearly impossible to resolve under current conditions and emphasizing that they could compromise patient safety in the home setting. Interviewees stressed that unless these barriers are adequately addressed, they could not only endanger the quality and safety of patient care, but also significantly reduce the willingness of healthcare professionals, especially physicians and nurses, to support the expansion of home treatment programs.

Beyond logistical challenges, interviewees also highlighted regulatory barriers that currently prevent the safe and efficient transition of certain treatments to the home setting. Approximately half of the physicians and nurses mentioned the need for regulatory revisions, such as modifying the requirement that two staff members be present during blood transfusions and advocating for the possibility of a single trained provider. Participants further emphasized the necessity of developing clear, standardized clinical protocols to guide decision-making during home-based care, noting that the absence of such protocols increases clinical uncertainty and undermines staff confidence in managing treatments outside the hospital environment.

*It requires regulatory changes or adjustments that could allow it to be done at home. (Physician 4).*

*Double verification is also something to consider, but again, it’s all a matter of certain regulations that we need to figure out how to implement……But first and foremost, clear protocols need to be written on how it should be done. (Nurse 8).*

#### Building communication and support structures to bridge safety concerns

As part of the solution to the concerns raised, there was a broad consensus among physicians and nurses about the need to establish a highly organized and structured home treatment system. Interviewees emphasized that such a system should operate comprehensively, with clear and detailed protocols covering all aspects of care. They stressed the importance of appointing a designated individual responsible for ensuring the system’s smooth and consistent operation.

A key component identified was the development of a centralized communication mechanism between patients and healthcare providers. Suggestions included appointing an intermediary to manage patient inquiries, ensuring organized and efficient communication. Participants proposed that this role be fulfilled by an external staff member dedicated to handling communication, ideally a nurse capable of triaging patient requests based on clinical urgency and providing initial guidance as needed. While most nurses did not anticipate major communication issues in a home treatment setting and supported continuing existing after-hours practices, namely, patients contacting physicians directly via mobile phone or email, or seeking emergency care at the hospital when needed, many physicians saw the transition to home care as an opportunity to formally restructure communication, expressing their exhaustion from receiving direct calls to their personal phones and advocating for a triage or screening system for incoming patient requests.

In addition, interviewees highlighted the necessity of setting clear expectations and maintaining open, structured communication with patients and their support networks, including obtaining their informed consent for receiving home-based treatment.

*I would prefer to have a coordinator whose sole job is to answer questions she is capable of handling, and for those she cannot, she gathers and organizes them. She should be skilled enough to distinguish between urgent and non-urgent issues, so that at the end of the day, you receive a set number of inquiries and respond accordingly. This does somewhat weaken the daily connection with patients, but the ability of a patient to intrude into your personal life at any given moment makes this profession much more challenging and significantly more exhausting. (Physician 3).*

### Theme 2: building a well-functioning home treatment system vs. fearing loss of control

#### The vision of a structured, high-quality, and patient-centered home treatment system

A major theme from the interviews was the utmost importance of assembling a highly skilled home treatment team. All participants agreed that the administration of treatments at home should be conducted by hemato-oncology nurses, given their expertise and familiarity with complex therapies. Physicians were expected to remain available on standby to provide clinical support if necessary. In situations where a patient’s condition deteriorated during home treatment, they suggested that the nurse would be responsible for calling emergency services and facilitating transfer to the hospital. However, approximately half of the nurses expressed major concern about the risks associated with being alone in a patient’s home during a medical emergency. They described this scenario as a substantial barrier to the successful implementation of home treatment services, highlighting issues related to clinical risk and emotional stress.

*The emphasis should be on having a consistent team that maintains communication with us… Ideally, it would be someone familiar with the patients, who knows how to work with them, and who has treated many similar cases. (Physician 6).*

*We’re talking about nurses going into patients’ homes, and they need to be highly experienced and very senior. (Nurse 3)*

Building upon these concerns, interviewees described the ideal home treatment system as a comprehensive, highly structured, and patient-centered model requiring careful coordination among multiple stakeholders. At the core of this vision stands a dedicated coordinator who would serve as the case manager for all referrals and communications. This individual would be responsible for overseeing the flow of information among all professionals involved in patient care, including physicians, nurses, pharmacists, administrators, and regulatory bodies, ensuring that each case is directed to the appropriate party and that collaboration is maintained throughout the treatment process. Interviewees emphasized that the coordinator’s role would extend beyond administrative functions to include active problem-solving, proactive management of complex situations, and facilitation of seamless communication across all components of the system.

Beyond the composition of the team and system coordination, interviewees discussed the responsibility of sustaining solid ties with the hospital. Two physicians emphasized that the involvement of the patient’s regular hospital physician in the home treatment process is essential, not only for providing clinical oversight but also for strengthening patient cooperation and trust. They believed that patients would be more willing to accept and adhere to home-based treatment if they perceived it as an extension of their relationship with their hospital physician, rather than as a separate or unfamiliar service. This connection was seen as vital for ensuring continuity of care, patient reassurance, and adherence to medical instructions.

*In my view, this should be a hospital-led initiative. The need should originate from hospitals, but the community and its support network must be involved very quickly. (Physician 4)*

*Look, again, the organization managing this must be the hospital. The doctor is based in the hospital, and the patient comes to see an oncologist there. The oncologist is the one who issues referrals, orders tests, and writes the entire medication protocol. So, in any case, this must be hospital-driven. That’s how I see it. (Nurse 3).*

Finally, regarding system ownership and operational responsibility, most physicians and nurses indicated that the home treatment program should be initiated and managed by hospitals, in collaboration with community healthcare services, but under the primary responsibility of the hospital itself. This model was seen as necessary to ensure high-quality standards, rapid access to hospital resources when needed, and continuous clinical oversight. Nevertheless, some interviewees proposed alternative models, suggesting that home treatments could instead be managed by health maintenance organizations (HMOs), using their existing infrastructure for community-based care. A few interviewees also mentioned the possibility of outsourcing home treatment services to private companies specializing in home healthcare, although this approach raised concerns among others about potential inconsistencies in quality and oversight.

Overall, the vision described by the interviewees portrays home treatment not as a simple relocation of hospital services to the patient’s home, but rather as the creation of a sophisticated, coordinated system that mirrors the complexity and professionalism of hospital-based treatments, while maintaining a strong emphasis on patient-centeredness, safety, and quality.

#### The promise of home-based care for enhancing patient well-being

Most interviewees believe that home treatment services offer significant potential to improve patients’ quality of life and reduce both physical and emotional strain associated with frequent hospital visits. These services were seen as especially beneficial in enhancing patient comfort, preserving autonomy, and minimizing disruptions to daily routines. While the advantages may apply broadly, interviewees noted that certain patients, such as those with strong home support systems, palliative care needs, limited mobility, or long travel distances to the hospital, might particularly benefit from receiving treatment in the home setting.

*I believe that in some cases, this could be beneficial for patients. It could prevent unnecessary hassle for very elderly or dependent patients who have difficulty coming here. (Nurse 6)*

#### Building a gradual transition model to maintain control and enable the ideal home treatment system

Considering the logistical, clinical, and regulatory challenges associated with transitioning treatments into the home setting, interviewees proposed a gradual, step-by-step approach as the preferred strategy for enabling safe and effective home-based treatments. According to participants, treatments should initially begin in the hospital, where patients can be closely monitored for adverse reactions. If the patient remains stable after several sessions and no severe side effects are observed, the treatment can then be safely continued at home.

Interviewees also suggested developing a hybrid model, in which patients alternate between hospital and home treatments, thereby reducing hospital visits while maintaining regular clinical oversight and contact with the medical team. Both physicians and nurses expressed consensus that this gradual transition model would increase the likelihood of success by allowing for risk management, building staff and patient confidence, and ensuring safety.

Participants highlighted that the first treatments selected for home administration should be those with low potential for harm and minimal logistical complexity. Physicians emphasized that starting with simpler cases would allow the system to develop experience and operational stability before expanding to more complex treatments.

*When it’s a regular treatment that’s not the first time the patient is receiving it, and we’ve seen that they haven’t had reactions, overall, it passes easily and it’s a short treatment. (Physician 8).*

*Perhaps initially, yes, to administer the first few times in a medical setting and then transfer it to home… other things too, if they give the first couple of times in a medical setting and see that there are no unusual reactions, then it’s safer to move to a home setting. (Nurse 1).*

### Theme 3: balancing costs, risks, and institutional support in home treatment implementation

#### Professional and patient trust as drivers of home treatment adoption

Interviewees emphasized the key role of medical staff and hospitals in the successful transition to home-based treatments. They suggested that physicians and nurses should play a central role in promoting home treatments among patients. Specifically, a physician and a nurse highlighted that the medical staff’s trust in the effectiveness and safety of home treatments is a critical factor influencing patient compliance. They described instances where patients failed to utilize available services simply because medical staff did not adequately communicate or promote these options, often due to a lack of confidence in their effectiveness.

Approximately half of the nurses indicated that endorsement by the Ministry of Health or evidence from prior successful implementations significantly increased their willingness to support home treatments. This was particularly apparent regarding home subcutaneous injections and supportive care services, which are already established practices.

*If this is something planned by the Ministry of Health, with all the necessary processes and possible scenarios in place, and I understand that there will be a staff member available to provide support, then I trust the Ministry of Health. (Nurse 6).*

Physicians explicitly addressed the transferability of treatments to home settings through considerations of risk management and cost–benefit analyses. In contrast, nurses did not directly discuss risk management or cost–benefit factors in their decisions regarding home treatments.

*A patient receiving blood products on a weekly or biweekly basis, who generally does not have reactions to them, I wouldn’t administer it at home for the first time, but if they typically don’t react, it seems like a reasonable approach from a general risk management perspective. (Physician 3).*

*I’m considering the question of cost-effectiveness, not just in terms of financial cost but also the personal cost, particularly in the relationship with the patient. The question is whether it’s worth it, because right now, the situation places a huge burden here… and that comes with its own costs, as the heavy workload makes it difficult to reach everyone. (Physician 6).*

#### Financial disincentives and organizational resistance to change

Economic considerations were discussed, including the budget necessary for staffing, recruitment, and the operation of home treatment systems. Some interviewees raised concerns regarding possible financial implications for hospitals, noting that hospitals might resist shifting treatments to patients’ homes due to the loss of revenue generated by patient visits. Physicians identified these economic considerations as a primary barrier delaying the transition of treatments to home care. In contrast, nurses addressed economic issues only from a practical viewpoint, noting the impracticality and inefficiency of dedicating extensive nursing hours, potentially an entire day, to individual patients in their homes, without referencing broader economic incentives or motivations behind institutional decisions.

To address these financial concerns, interviewees recommended convincing hospitals to get involved in the development and provision of home treatments, suggesting that this could be facilitated through public relations efforts and the adoption of a patient-centered care approach.

*I think many people have interests in not overcoming the technical problems… it’s a financial issue, it’s all about money. (Physician 3)*

*If hospitals were incentivized for treating patients at home, they would no longer have an interest in bringing them in. There would be no reason for patients to come here. Right now, something about the financial incentives doesn’t align with the broader goals. (Physician 3)*

## Discussion

This study explored physicians’ and nurses’ perceptions of home-based treatment for hematology and hemato-oncology patients, identifying key factors shaping their attitudes toward this treatment approach. The results indicate a multifaceted interaction between clinical concerns, organizational barriers, and professional beliefs, emphasizing the potential benefits and the challenges associated with transitioning care from hospital to home settings.

A central tension identified in the study was the perception of hospitals as “safe havens” and highly controlled environments where adverse reactions, complications, and deterioration can be immediately managed. Participants, particularly nurses, expressed considerable anxiety about the diminished ability to monitor patients and intervene promptly once care is transferred to the home. These concerns match with previous literature on home-based care for complex patients, which also emphasizes the risks associated with fragmented communication, medication management challenges, and coordination failures following hospital discharge [14, 17].

However, despite these safety concerns, many participants acknowledged that home-based treatment increases patient comfort and potentially improves their quality of life, aligning with broader trends in healthcare policy. The findings demonstrate that clinical endorsement of home-based care is conditional; while physicians and nurses recognize its benefits, their support is dependent on the establishment of robust systems to mitigate risks. Participants also did not view all home care methods as equally feasible. Consistent with the findings, low-risk interventions such as supportive care services, subcutaneous therapies, and treatments that were already safely administered in hospital settings were generally seen as more appropriate for home care. In contrast, blood transfusions and more complex treatments were seen as requiring greater caution, additional infrastructure, and closer clinical supervision. These findings support a phased implementation strategy in which low-risk interventions serve as an entry point to broader home hemato-oncology services.

Participants described several patient-centered benefits of home care, including increased convenience, reduced travel burden, the ability to maintain a daily routine, and the ability to remain close to family and support networks. A small number of participants also emphasized the potential reduction in exposure to hospital-associated infections, an issue relevant to immunocompromised hemato-oncology patients. Interestingly, although participants recognized these potential benefits, they paid greater attention to safety, organizational preparedness, and risk management. This emphasis likely reflects their professional responsibility for ensuring patient safety and the fact that most participants were clinicians working in hospitals.

One such system, repeatedly emphasized across interviews, is the establishment of a dedicated coordination role. The need for a central figure responsible for linking hospital and home-based services, managing communication among professionals, and ensuring continuity of care reflects broader calls in the literature for structured coordination mechanisms in home healthcare [13, 18]. Participants viewed coordination not merely as an operational convenience but as a critical safety net that could help preserve the clinical standards associated with hospital-based care.

The findings also highlight the importance of collaboration between hospitals and community health services. Although routine home hemato-oncology care remains limited in Israel, community health infrastructures, including home care services operated by health maintenance organizations (HMOs) and private healthcare companies, may provide a basis for future implementation. Participants often envisioned a collaborative model in which hospitals would provide clinical oversight while community services would support care delivery and ongoing patient follow-up.

Importantly, the findings highlight that attitudes toward home-based treatment are shaped not only by clinical and logistical considerations but also by institutional and financial factors. These findings align with a recent study [19] that emphasized that effective transitions require coordinated actions ranging from personalized discharge planning to institutional accountability and national resource allocation. This policy-oriented approach aligns with our participants’ views that, without system-wide support, the transition to home-based treatment is unlikely to succeed, even if clinically feasible.

Physicians identified financial disincentives, such as potential revenue losses for hospitals, as significant barriers to system-level change. While nurses focused more on the practical challenges of dedicating extensive time to individual patients, both groups recognized that without institutional commitment and realignment of economic incentives, home treatment models are unlikely to be sustainably integrated into healthcare systems.

While prior implementation research emphasizes the importance of healthcare professionals’ self-efficacy, attitudes, and motivation in facilitating the adoption of innovations [20], this research adds to the literature by highlighting the perceived reciprocal impact of professional conviction on patient acceptance. Participants explicitly recognized that their own trust in home-based care models was a direct determinant of patients’ willingness to engage with such services, underscoring the critical interpersonal dimension of clinical innovation implementation.

Based on the findings, four key domains emerged as central to supporting the safe and effective implementation of home-based care for hemato-oncology patients: the need for structured coordination mechanisms, development of clinical and regulatory frameworks, targeted staff training, and alignment of institutional and financial incentives. These thematic areas reflect both the core concerns raised by participants and the practical conditions required to enable a sustainable shift from hospital-based to home-based treatment models.

Within these domains, several concrete and actionable steps are recommended:

1. Appoint a dedicated coordinator responsible for overseeing referrals, managing communication between stakeholders, and ensuring continuity of care.
2. Develop standardized clinical protocols for home-based administration of treatments, including cytotoxic drugs and blood transfusions.
3. Revise regulatory requirements to support flexible staffing models (e.g., allowing single-provider blood transfusions under specific conditions).
4. Ensure logistical infrastructure for safe medication storage, transportation, and equipment availability in the home setting.
5. Initiate targeted training programs to prepare nurses and physicians for clinical, logistical, and emergency scenarios in the home environment.
6. Design a gradual transition model, starting with low-risk treatments and patients with stable clinical profiles.
7. Maintain a hybrid care approach, combining home- and hospital-based treatments to support oversight and patient confidence.
8. Promote involvement of hospital-based physicians in the planning and supervision of home care to keep trust and continuity.
9. Align financial incentives to support hospital and provider engagement in home-based models.
10. Build organizational support and culture change by demonstrating the value of home care in enhancing patient experience and system efficiency.

Future research should explore how healthcare professionals’ attitudes toward home treatment evolve over time, particularly as home-based treatment becomes more established and familiar. Longitudinal studies could provide insights into whether exposure and experience reduce initial hesitations. Additionally, incorporating patient and family perspectives alongside provider views would be valuable for designing integrated, patient-centered home care models that meet the needs and expectations of all stakeholders. Finally, empirical evaluation of the clinical outcomes, patient safety indicators, and economic impacts of home-based initiatives will be crucial for informing sustainable policy development and funding strategies.

### Strengths and limitations

Qualitative design allowed for an in-depth exploration of attitudes, hesitations, and perceived barriers, generating rich contextual insights essential for guiding policy development and service design. One potential limitation is the risk of selection bias, as participants may have held particularly strong views about home-based treatment, potentially affecting the representativeness of the findings. However, this risk was mitigated by the recruitment process; interviews were conducted on randomly selected days, and all available staff members present in the department at the time were invited to participate. No participants refused to be interviewed, reducing the likelihood that the sample disproportionately represented individuals with extreme opinions. Nonetheless, as all participants were hospital-based, there is a potential for inherent bias, such as a tendency to view hospital-led models as the default or preferred framework for care. Additionally, most participants had no direct experience delivering home-based hemato-oncology care. While some were familiar with home-based care models through professional exposure or knowledge of existing initiatives, for others, the concept was relatively new. As a result, some of the views expressed may reflect anticipated opportunities and concerns rather than experiences derived from direct involvement in home-based care programs.

The relatively short duration of the interviews may be considered a limitation. However, the interviews were deliberately designed to focus on specific organizational, logistical, and clinical aspects of home care rather than personal experiences or emotionally oriented narratives. Participants provided focused responses, and recurring themes emerged consistently throughout the interviews, supporting the richness and relevance of the data collected.

Beyond mitigating concerns about selection bias, the study’s inclusion of both physicians and nurses enabled the capture of a broad spectrum of professional perspectives, highlighting both interprofessional differences and shared concerns that might otherwise have been overlooked. Furthermore, by addressing a timely and evolving issue at a time when healthcare systems are increasingly exploring home-based treatment models, the findings offer relevant and actionable insights for current discussions about service transformation.

Finally, a key contribution of the study lies in identifying the perceived link between healthcare professionals’ belief in the home treatment model and patients’ willingness to accept it. This finding points out the critical role of professional endorsement in the successful implementation of home-based treatment and highlights the need for future efforts to actively engage healthcare providers as partners in service innovation.

## Conclusion

This study offers important insights into the perceived challenges and attitudes of physicians and nurses toward home-based treatment for hematology and hemato-oncology patients. While many participants expressed support for the model, their perspectives were shaped by concerns about patient safety, logistical complexity, and systemic limitations. Addressing these challenges will require policy reform, training, and coordinated care structures. Ultimately, the success of home-based care depends not only on logistical feasibility but also on the active engagement and confidence of the healthcare professionals delivering it.

## Supplementary information

Below is the link to the electronic supplementary material.

ESM 1 (DOCX 27.4 KB)

## Data Availability

The datasets generated and analyzed during the current study (i.e., interview transcripts) are not publicly available due to confidentiality agreements with participants. Anonymized excerpts supporting the findings are available from the corresponding author upon reasonable request. The interview guide used in this study is included as a supplementary file.
