# Supplementary material for: Bringing the hospital home: exploring the challenges and perspectives of healthcare professionals on home treatment in hemato-oncology: a qualitative analysis
Source: Support Care Cancer. 2026 Jul 23;34(8):793. doi: 10.1007/s00520-026-10981-8 (PMC13396004; doi:10.1007/s00520-026-10981-8)
Supplement: Supplementary file 1 — (DOCX 27.4 KB) [file 520_2026_10981_MOESM1_ESM.docx]

**Supplementary material**

Additional file 1

Guide to a semi-structured interview

1. Please tell me about your role in working with hemato-oncology patients.
2. In your opinion, which treatments can be transferred from the hospital setting and provided at the patient’s home?
3. Why do you think these treatments are suitable for home-based care?
4. What challenges or problems might arise in delivering treatments at home?
5. What factors could support the successful transition of these treatments to the home setting?
6. Which treatments do you believe cannot be provided at home, and why?
7. Which healthcare professionals do you think should deliver care in the patient’s home?
8. How is continuity of care currently maintained between the patient and hospital-based medical staff?
9. What do you think is the best way to establish effective communication between the patient at home and the hospital medical team?
10. In your opinion, what would be the appropriate steps and frameworks for establishing such a home-based care service?

Additional file 2

COREQ report

| No | Item | description |
| --- | --- | --- |
| Domain 1: Research team and reflexivity | | |
| 1 | Interviewer/facilitator | The interviews were conducted by three authors (EE, MHA, YD). |
| 2 | Credentials | One interviewer (EE) is a PhD candidate in Public Health, the second interviewer (MHA) was a final-year MPH graduate student. The third interviewer held an MPH degree. |
| 3 | Occupation | At the time of the study, one interviewer (EE) was employed as a research assistant in the field of public health, while the second interviewer was working as a pharmacist. The third interviewer was employed as a research assistant in the field of public health. |
| 4 | Gender | All three interviewers are female. |
| 5 | Experience and training, Relationship with participants | All three interviewers had prior experience conducting in-depth qualitative interviews. In addition, each interviewer had completed one or two graduate-level courses in qualitative research methods as part of their advanced academic training. |
| 6 | Relationship established | No prior relationship existed between the interviewer and the participants before the study commenced. |
| 7 | Participant knowledge of the interviewer | Participants were informed about the researcher’s academic affiliation and the general aim of the study. |
| 8 | Interviewer characteristics | The research team reflected on potential preconceptions during the analytic process through regular peer debriefing. |
| Domain 2: study design | | |
| 9 | Methodological orientation and Theory | Reflexive Thematic Analysis (Braun & Clarke). |
| 10 | Sampling | An opportunistic sampling approach was used. All eligible healthcare staff who were present during their work shifts at the time of data collection were invited to participate in the study. |
| 11 | Method of approach | Participants were primarily approached face-to-face during their work shifts. A small number of interviews were conducted via Zoom, following recruitment by the principal investigator. |
| 12 | Sample size | A total of 23 participants took part in the study. |
| 13 | Non-participation Setting | No eligible participants who were approached refused to participate. Some potential participants were not available at the time of approach due to clinical workload (physicians and nurses). Recruitment at each institution ceased once the predetermined target number of interviews had been reached and thematic saturation was achieved |
| 14 | Setting of data collection | Most interviews were conducted face-to-face in the hospital wards. Three interviews were conducted via Zoom at a time convenient for the participants. |
| 15 | Presence of non-participants | No one else was present during the interviews besides the participant and the researcher. |
| 16 | Description of sample Data collection | The study population included 23 participants: 11 physicians and 12 nurses from eight hospitals across Israel. The physicians included four men and seven women, ranging in seniority from residents to department chairs. The nursing staff included one male and eleven female nurses, with varying levels of professional experience, from relatively new staff members to senior managerial head nurses. Participants were recruited from hospitals of different sizes and from diverse geographic regions, including northern, southern, central Israel, and the Jerusalem area. |
| 17 | Interview guide | A semi-structured interview guide was developed by the authors and used for all interviews. A formal pilot study was not conducted; however, minor iterative refinements to the interview guide were made throughout the data collection process based on ongoing reflection and emerging insights from the interviews. |
| 18 | Repeat interviews | No repeat interviews were conducted. |
| 19 | Audio/visual recording | All interviews were audio-recorded for transcription. |
| 20 | Field notes | Field notes were taken during the interviews. |
| 21 | Duration | Interviews lasted between 8 and 35 minutes, with a mean duration of 21 minutes. |
| 22 | Data saturation | Data saturation was discussed and agreed upon by the research team during the analytic process. |
| 23 | Transcripts returned | Transcripts were not returned to participants for comment or correction. |
| Domain 3: analysis and findings | | |
| 24 | Number of data coders | The primary coding of the data was conducted by a single researcher; however, analytic decisions and theme development were discussed regularly with the research team throughout the analysis process. |
| 25 | Description of the coding tree | No, a formal coding tree was not explicitly presented. The coding process and theme development were described narratively in the Methods and Results sections. |
| 26 | Derivation of themes | Themes were generated inductively from the data using a reflexive thematic analysis approach, rather than being defined a priori. |
| 27 | Software | No qualitative data analysis software was used; the interview transcripts were managed and coded manually |
| 28 | Participant checking Reporting | No, participants did not provide feedback on the study findings. Morever, two co-authors (AA and AS), who are physicians and represent the medical teams within the research team, contributed to the interpretation of findings, providing clinical perspectives during the analytic process. |
| 29 | Quotations presented | Yes, participant quotations are presented to illustrate the themes and findings. Each quotation was identified by both professional role (physician or nurse) and participant number. |
| 30 | Data and findings, consistent | Yes, there was clear consistency between the data presented and the study’s findings, with participant quotations appropriately supporting the identified themes. |
| 31 | Clarity of major themes | Yes, the major themes were clearly presented in the findings. |
| 32 | Clarity of minor themes | Yes, minor themes (sub-themes) were discussed within each major theme; however, a formal analysis of diverse or negative cases was not explicitly presented. |

Additional file 3

Summary of results

| **Theme** | **Sub-theme** | **Description** | **Quote** |
| --- | --- | --- | --- |
| 1. The hospital as a safe haven: The Conflict Between Hospital Safety and the benefits of Home Treatment | 1.1 The Hospital as a Stronghold of Safety and Clinical Supervision | The hospital was viewed as a stronghold of safety, enabling immediate response and continuous clinical supervision. | *"Look, sometimes when a patient comes here and I take a brief medical history, if they have a cold, they won’t really be able to hide it from me. I’ll see it, I’ll hear it. If they seem a bit unsteady, I’ll notice. But if they’re at home and don’t want to skip the treatment, I’ll talk to them on the phone, and take their medical history over the phone, but I won’t pick up on it." (Nurse 5)* |
|  | 1.2 Concerns about Fragmentation, Risk, and Unmanageable Complexity | Interviewees identified significant logistical and regulatory barriers to safe home treatment, including medication handling, equipment needs, and complex treatment management. They emphasized that unresolved barriers could compromise patient safety and reduce healthcare professionals' willingness to support home-based treatments. | *"Logistically, regarding medication supply, there are extremely expensive medications. If it spills on the way, if out of 40 grams of IgG, 10 grams are spilled because the patient didn't manage it properly or didn't store it in the right conditions, placed it by a window near the sun, then it's a terrible loss, it's a shame…...If 80 patients need to receive IgG today, a nurse needs to be with them, you need 80 nurses to be with the patients at home, where will you find them?". (Physician 7)* |
|  | 1.3 Building Communication and Support Structures to Bridge Safety Concerns | Staff emphasized the need for a highly organized home treatment system with clear protocols and a designated coordinator, alongside a centralized communication mechanism to manage patient inquiries, set expectations, and ensure informed consent. | *"The patient needs to feel that they have support, especially during treatments, and this is not trivial. When they are in day hospitalization, they are surrounded by care. Currently, communication is limited to "we have an emergency department here," etc., but only during working hours. If such a system is established, it should function like in the U.S., with a dedicated system that receives calls and directs them to physicians, ensuring there is an on-call doctor. If a patient feels unwell, they should know where to turn and receive a response. Right now, it mostly depends on the specific doctor and how available and willing they are to respond." (Physician 4)* |
| 1. Building a Well-Functioning Home Treatment System vs. Fearing Loss of Control | 2.1 The Vision of a Structured, High-Quality, Patient-Centered Home Treatment System | Interviewees described the ideal home treatment system as a highly structured, hospital-led service, coordinated by a dedicated case manager and staffed by skilled oncology nurses. They emphasized that strong integration with the hospital is essential to maintain direct medical responsibility, strengthen patient trust, and ensure seamless, high-quality care. | *"A complete system is needed that can support this. In other words, it's not just about bringing the bag home and that's it... there needs to be someone who coordinates, someone responsible, someone who handles issues and knows how to respond to them." (Physician 4)* |
|  | 2.2 The Promise of Home-Based Care for Enhancing Patient Well-Being | Staff identified specific patient groups, such as palliative, remote, and mobility-challenged patients, for whom home-based care could ease physical strain and enhance quality of life. | *"There are patients for whom blood product administration is an integral part of the protocol because their counts drop significantly. I have no interest in having them come here, it endangers them, it's a hassle for them because they don't feel well after treatment, and therefore, if it were possible to provide this at home, it would be very helpful." (Physician 3)* |
|  | 2.3 Building a Gradual Transition Model to Maintain Control and Enable the Ideal Home Treatment System | Interviewees suggested a range of strategies centered on a gradual, step-by-step transition to home treatment, including starting treatments in the hospital, monitoring patient stability, and using hybrid models to maintain clinical control, manage risks, and support the successful implementation of the ideal system. | *"If I were to do something like this, I would do it gradually. That is, starting with those whose potential risk of harm is lower." (Physician 3)* |
| 1. Balancing Costs, Risks, and Institutional Support in Home Treatment Implementation | 3.1 Professional and Patient Trust as Drivers of Home Treatment Adoption | Interviewees emphasized that medical staff trust in the safety and effectiveness of home treatments, along with institutional endorsement, is crucial for patient acceptance. Physicians additionally considered risk management and cost-benefit factors when evaluating home treatment transferability. | *"It should also have the approval and endorsement of the treating physician, as this is a very important aspect. If the physician recommends, I'm not saying convinces, but presents the positive perspective, it can greatly influence the patient by highlighting the benefits." (Nurse 5)* |
|  | 3.2 Financial Disincentives and Organizational Resistance to Change | Interviewees identified financial disincentives and potential revenue loss for hospitals as major barriers to the adoption of home treatment, suggesting that aligning institutional incentives and promoting a patient-centered approach could facilitate change. | *"The key to success is first and foremost making hospitals understand that they want this, using financial incentives, public relations leverage, and a patient-centered care approach. These are the main drivers of change in the system. When something is economically viable and contributes to the hospital’s image, not just its image, but its philosophy, it makes the institution more attractive." (Physician 4)* |
